# Twitter Discussions and Emotions About the COVID-19 Pandemic: Machine Learning Approach

**DOI:** 10.2196/20550

**Published:** 2020-11-25

**Authors:** Jia Xue, Junxiang Chen, Ran Hu, Chen Chen, Chengda Zheng, Yue Su, Tingshao Zhu

**Affiliations:** 1 Factor-Inwentash Faculty of Social Work University of Toronto Toronto, ON Canada; 2 Faculty of Information University of Toronto Toronto, ON Canada; 3 School of Medicine University of Pittsburgh Pittsburgh, PA United States; 4 Middleware System Research Group University of Toronto Toronto, ON Canada; 5 CAS Key Laboratory of Behavioral Science Institute of Psychology Chinese Academy of Sciences Beijing China; 6 Department of Psychology University of Chinese Academy of Sciences Beijing China

**Keywords:** machine learning, Twitter data, COVID-19, infodemic, infodemiology, infoveillance, public discussion, public sentiment, Twitter, social media, virus

## Abstract

**Background:**

It is important to measure the public response to the COVID-19 pandemic. Twitter is an important data source for infodemiology studies involving public response monitoring.

**Objective:**

The objective of this study is to examine COVID-19–related discussions, concerns, and sentiments using tweets posted by Twitter users.

**Methods:**

We analyzed 4 million Twitter messages related to the COVID-19 pandemic using a list of 20 hashtags (eg, “coronavirus,” “COVID-19,” “quarantine”) from March 7 to April 21, 2020. We used a machine learning approach, Latent Dirichlet Allocation (LDA), to identify popular unigrams and bigrams, salient topics and themes, and sentiments in the collected tweets.

**Results:**

Popular unigrams included “virus,” “lockdown,” and “quarantine.” Popular bigrams included “COVID-19,” “stay home,” “corona virus,” “social distancing,” and “new cases.” We identified 13 discussion topics and categorized them into 5 different themes: (1) public health measures to slow the spread of COVID-19, (2) social stigma associated with COVID-19, (3) COVID-19 news, cases, and deaths, (4) COVID-19 in the United States, and (5) COVID-19 in the rest of the world. Across all identified topics, the dominant sentiments for the spread of COVID-19 were anticipation that measures can be taken, followed by mixed feelings of trust, anger, and fear related to different topics. The public tweets revealed a significant feeling of fear when people discussed new COVID-19 cases and deaths compared to other topics.

**Conclusions:**

This study showed that Twitter data and machine learning approaches can be leveraged for an infodemiology study, enabling research into evolving public discussions and sentiments during the COVID-19 pandemic. As the situation rapidly evolves, several topics are consistently dominant on Twitter, such as confirmed cases and death rates, preventive measures, health authorities and government policies, COVID-19 stigma, and negative psychological reactions (eg, fear). Real-time monitoring and assessment of Twitter discussions and concerns could provide useful data for public health emergency responses and planning. Pandemic-related fear, stigma, and mental health concerns are already evident and may continue to influence public trust when a second wave of COVID-19 occurs or there is a new surge of the current pandemic.

## Introduction

Thirty million cases of COVID-19 have been confirmed across 110 countries as of mid-September 2020, and the death toll has reached close to 947,000 [1]. The widespread use of social media, such as Twitter, accelerates the process of exchanging information and expressing opinions about public events and health crises [2-5]. COVID-19 has been one of the trending topics on Twitter since January 2020 and has continued to be discussed to date. Since quarantine measures have been implemented across most countries (eg, the shelter-in-place order in the United States), people have been increasingly relying on different social media platforms to receive news and express opinions. Twitter data are valuable for revealing public discussions and sentiments related to various topics, as well as real-time news updates during global pandemics, such as H1N1 and Ebola [6-9]. Chew and Eysenbach’s study [6] showed that Twitter could be used for real-time “infodemiology” studies, providing a source of opinions for health authorities to respond to public concerns. During the COVID-19 pandemic, many government officials worldwide have used Twitter as one of their main communication channels to regularly share policy updates and news related to COVID-19 to the general public [10].

Since the COVID-19 outbreak, a growing number of studies have collected Twitter data to understand the public responses to and discussions around COVID-19 [11-18]. For instance, Abd-Alrazaq and colleagues [11] adopted topic modeling and sentiment analysis to determine the main discussion themes and sentiments around COVID-19, using tweets collected between February 2 and March 15, 2020. Budhwani and Sun [14] compared Twitter discussions before and after March 16, 2020, when President Trump tweeted about the “Chinese virus,” and found a significantly increased use of the phrase “Chinese virus” in people’s tweets across many US states afterward. Mackey and colleagues [16] analyzed about 3465 tweets collected between March 2 and 20, 2020, using a topic model to explore users’ self-reported experiences with COVID-19 and related symptoms. Ahmed and colleagues [12] conducted social network analysis and content analysis of collected tweets between March 27 and April 4, 2020, to understand what may have driven the misinformation that linked 5G towers in the United Kingdom to the COVID-19 pandemic. As conversations on Twitter continue to take place and evolve, it is worth continuing to use tweets as a source of data to track and understand the salient topics discussed on Twitter in response to the COVID-19 pandemic and track their changes across time.

To expand the literature on public reactions to the COVID-19 pandemic, this study aims to examine the public discourse and emotions related to the COVID-19 pandemic by analyzing more than 4 million tweets collected between March 7 and April 21, 2020.

## Methods

### Research Design

**Figure 1:**
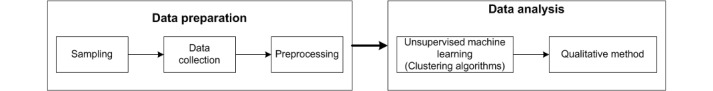
Twitter data mining pipeline.

We used a purposive sampling approach to collect COVID-19–related tweets published between March 7 and April 21, 2020. Our Twitter data mining approach followed the pipeline displayed in Figure 1. Data preparation included the following three steps: (1) sampling, (2) data collection, and (3) preprocessing the raw data. The data analysis stage included unsupervised machine learning, sentiment analysis, and thematic qualitative analysis*.* The unit of analysis was each message-level tweet. Unsupervised learning is one approach in machine learning; it is used to examine data for patterns, and derives a probabilistic clustering based on text data. We chose unsupervised learning because it is commonly used when existing studies have few observations of or insights into unstructured text data [19]. Since a qualitative approach would be challenging when analyzing large-scale Twitter data, unsupervised learning allows us to conduct exploratory analyses of large text data for social science research. In this study, we first employed an unsupervised machine learning approach to identify salient latent topics. We used a thematic analysis approach to develop themes further, allowing a deeper dive into the data, such as through manual coding and inductively developing themes based on the latent topics generated by machine learning algorithms.

### Sampling and Data Collection

**Figure 2:**
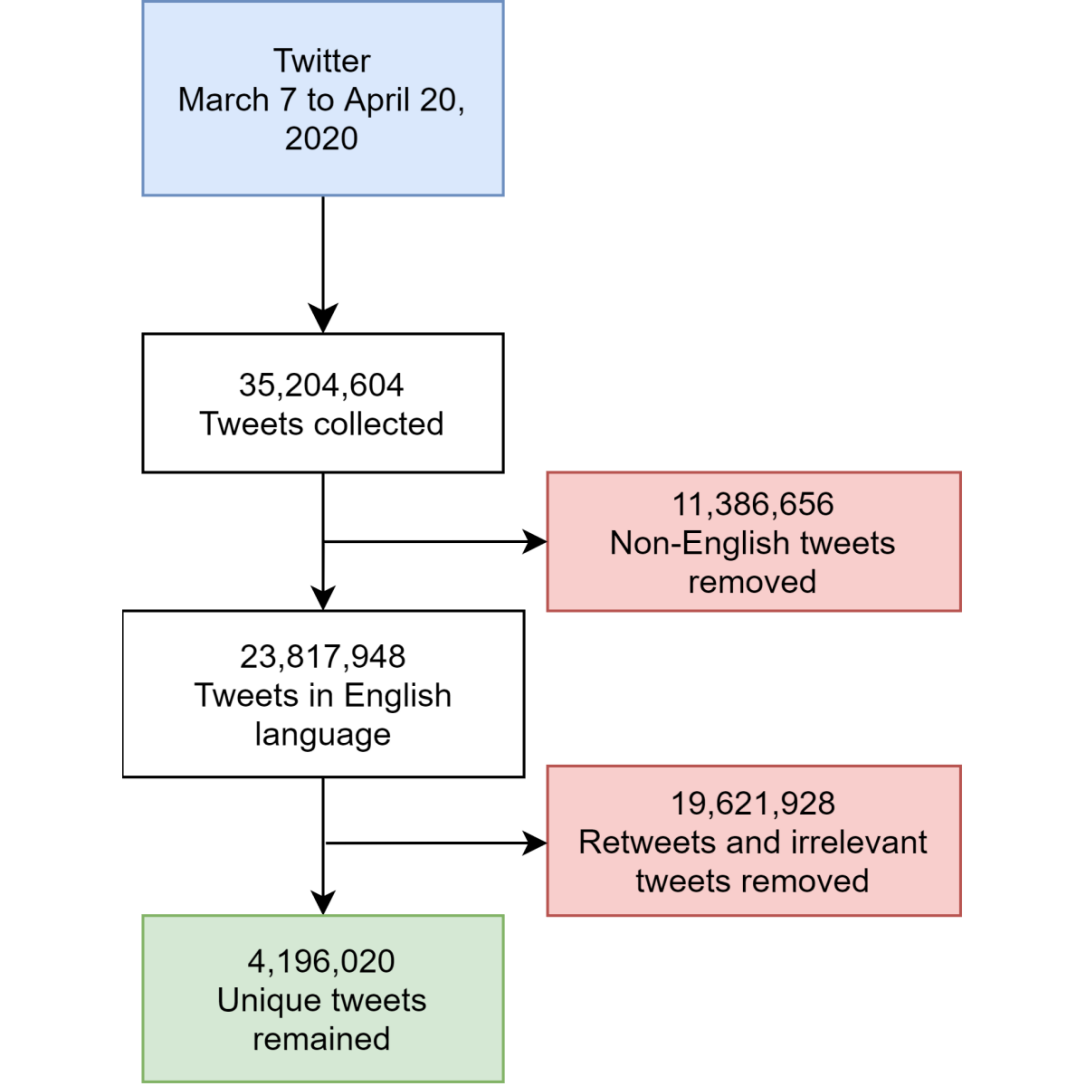
Tweet preprocessing chart.

We used a list of COVID-19–related hashtags as search terms to fetch tweets (eg, #coronavirus, #2019nCoV, #COVID19, #coronaoutbreak, and #quarantine; Multimedia Appendix 1). Twitter’s open application programming interface (API) allowed us to collect updated Twitter messages set to open by default. From March 7 to April 21, 2020, we collected 35,204,604 tweets during this period (Figure 2). After removing non-English tweets, 23,817,948 tweets remained. After removing duplicates and retweets (ie, tweets that only repost the original message without adding any more words), we had 4,196,020 tweets in our final data set. We collected and downloaded the following features for each tweet: (1) the full text, (2) the numbers of favorites, followers, and followings, (3) users’ geolocation, and (4) users' description/self-created profile.

### Preprocessing the Raw Data

We used Python to clean the raw data (Figure 1). The process was as follows [18]:

1. We removed the hashtag symbol, @users, and URLs from the tweets in the data set.
2. We removed non-English characters (non-ASCII characters) because this study focused on tweets in English.
3. We removed special characters, punctuation, and stop-words [19] from the data set as they do not contribute to the semantic meanings of messages.

### Data Analysis

#### Unsupervised Machine Learning

Latent Dirichlet Allocation (LDA) [20] is a widely used unsupervised machine learning approach that allows researchers to analyze unstructured text data (eg, Twitter messages). Based on the data itself, the algorithm produces frequently mentioned pairs of words, the pairs of words that co-occur together, and latent topics and their distributions over topics in the document [21]. Existing studies have indicated the feasibility of using LDA to identify the patterns and themes of tweets related to COVID-19 [11,22].

#### Qualitative Analysis

To triangulate and contextualize findings from the LDA model, we employed a qualitative approach to develop themes further. Specifically, we used Braun and Clarke's [23] six steps of thematic analysis: (1) getting familiar with the keyword data, (2) generating initial codes, (3) searching for themes, (4) reviewing potential themes, (5) defining themes, and (6) reporting. In addition to following the six-phase approach, our process was iterative and reflective by moving backward and forward through the six phases [24]. The thematic approach relied on human interpretation, a process that can be significantly influenced by personal understanding of the topics and a variety of biases. Two team members who have experience analyzing Twitter data documented their thoughts about potential codes in NVivo independently. Two other team members then reviewed the initial codes and considered whether they reflected the identified topics. For example, two team members collapsed several similar codes into one theme to ensure the topics corresponded meaningfully under one theme. The next stage was naming the themes to ensure the themes fitted into the overall meanings of the identified salient topics. We finalized themes corresponding to each of the 13 topics.

#### Sentiment Analysis

We used sentiment analysis, a natural language processing (NLP) approach, to classify the main sentiments of a given twitter message, such as fear and joy [25]. In this study, we used the NRC Emotion Lexicon, which consists of 8 primary emotions: anger, anticipation, fear, surprise, sadness, joy, disgust, and trust [26]. We followed 4 steps to calculate the emotion index for each Twitter message: (1) removed articles and pronouns (eg, “and,” “the,” “to”), (2) applied a stemmer by removing the predefined list of prefixes and suffixes (eg, “running” becomes “run” after stemming) [27], and (3) calculated the emotion index (if a sentence had multiple emotions, we only kept the emotion with the highest matching count), and (4) calculated the scores for each 8-emotion type. We discussed these 4 steps in detail in a previous study [18].

## Results

### Descriptive Results

**Figure 3:**
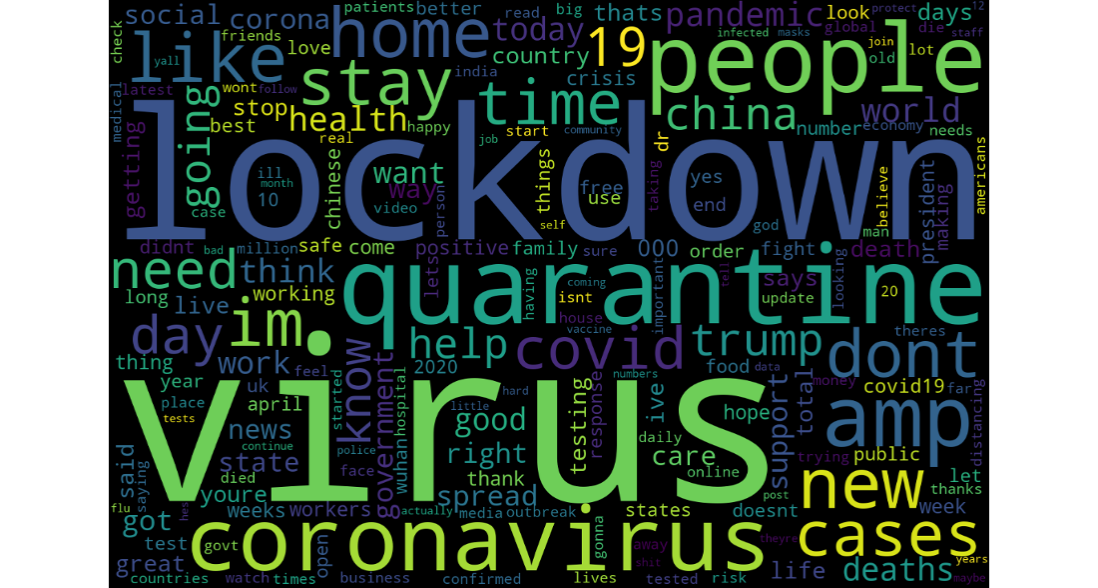
The word cloud of the most popular unigram.

**Figure 4:**
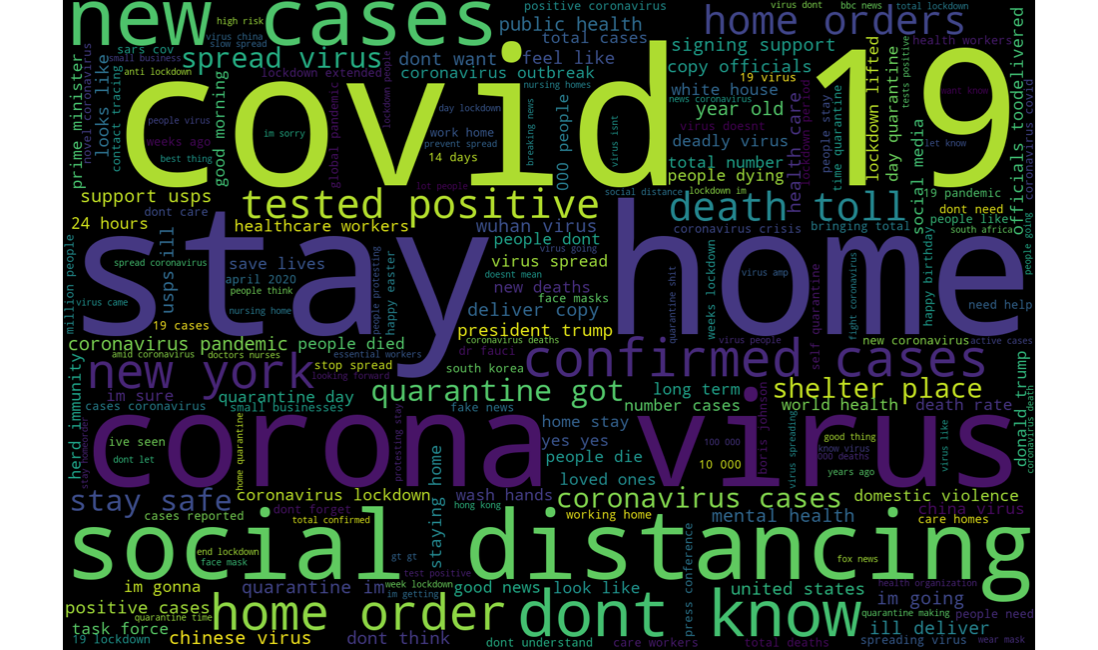
Word cloud of the most popular bigrams.

In total, after preprocessing all raw data, our final data set included 4,196,020 tweets. We identified the most popular tweeted bigrams (pairs of words) related to COVID-19. Bigrams captured “two concessive words regardless of the grammar structure and semantic meaning and may not be self-explanatory” [21]. Bigrams identified included the following: “covid 19,” “stay home,” “social distancing,” “new cases,” “don't know,” “confirmed cases,” “home order,” “New York,” “tested positive,” “death toll,” and “stay safe.” Popular unigrams included “virus,” “lockdown,” “quarantine,” “people,” “new,” “home,” “like,” “stay,” “don't,” and “cases.” We presented the most popular unigrams and bigrams related to COVID-19 in Table 1 and visualized them using word clouds in Figures 3 and 4.

**Table 1:** Top 50 bigrams and unigrams and their distributions.

Top 50 bigrams	Percentage of data set	Top 50 unigrams	Percentage of data set
covid 19	0.29	virus	1.18
stay home	0.26	lockdown	0.98
corona virus	0.12	quarantine	0.94
social distancing	0.08	people	0.82
new cases	0.07	coronavirus	0.79
dont know	0.04	new	0.47
confirmed cases	0.04	home	0.45
home order	0.04	like	0.44
new york	0.04	im	0.41
tested positive	0.04	stay	0.41
death toll	0.04	dont	0.41
home orders	0.04	cases	0.37
quarantine got	0.03	time	0.36
stay safe	0.03	covid	0.35
spread virus	0.03	19	0.30
coronavirus cases	0.03	need	0.30
shelter place	0.03	day	0.29
coronavirus pandemic	0.03	trump	0.28
year old	0.03	china	0.28
public health	0.03	know	0.28
chinese virus	0.03	going	0.25
ill deliver	0.03	help	0.25
deliver copy	0.03	pandemic	0.24
health care	0.03	world	0.24
support usps	0.03	health	0.23
signing support	0.02	think	0.22
usps ill	0.02	deaths	0.21
wuhan virus	0.02	today	0.21
quarantine im	0.02	good	0.20
mental health	0.02	work	0.20
dont want	0.02	want	0.19
im going	0.02	corona	0.17
president trump	0.02	spread	0.17
united states	0.02	got	0.17
dont think	0.02	support	0.17
copy officials	0.02	government	0.17
feel like	0.02	right	0.15
looks like	0.02	way	0.15
positive cases	0.02	care	0.15
staying home	0.02	social	0.15
officials toodelivered	0.02	news	0.15
coronavirus outbreak	0.02	state	0.15
domestic violence	0.02	country	0.15
coronavirus lockdown	0.02	said	0.14
healthcare workers	0.02	ive	0.14
people died	0.02	days	0.14
quarantine day	0.02	testing	0.14
donald trump	0.02	stop	0.13
social media	0.02	says	0.13

### COVID-19–Related Topics

**Figure 5:**
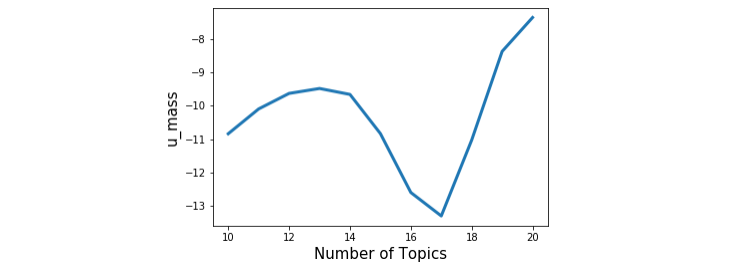
The number of topics based on the coherence model.

Our approach, LDA, produced frequently co-occurring pairs of words related to COVID-19 and organized these co-occurring words into different topics. LDA allowed us to manually define the number of topics (eg, 10 topics, 20 topics) that we would like to generate. Consistent with previous studies, we used the coherence model, Gensim (RARE Technologies Ltd) [28], to calculate the most appropriate number of topics based on the data itself. For this data set, the LDA indicated that having 13 topics would give a high coherence score and the smallest topic number (eg, while having 19 or 20 topics would give a higher coherence score, they involve more topics; Figure 5).

We further analyzed the document-term matrix and obtained the distributions of 13 topics. We presented the results of 13 salient topics and the most popular pairs of words (bigrams) within each topic in Table 2. For example, Topic 3 had the highest distribution (8.87%) among all 13 common latent topics. The bigrams associated with Topic 3 included “tested positive,” “coronavirus outbreak,” “New York,” “shelter place,” and “mental health.” These pairs of words frequently co-occurred together, and therefore the LDA model assigned them to the same topic.

**Table 2:** Identified salient topics, bigrams, and their distributions.

Topic	Bigrams within topics	Distribution (%)
1	covid 19, dont know, deadly virus, im gonna, spreading virus, 19 lockdown, herd immunity, 000 people,19 pandemic, dont need, face masks, fox news, health workers, small businesses, home quarantine, like this, virus came, slow spread, test kits, total confirmed	8.51
2	spread virus, health care, staying home, white house, positive cases, people die, 14 days, coronavirus deaths, care workers, ive seen, need help, day lockdown, know virus, im getting, doctors nurses, quarantine period, virus world, stop virus, people getting, week quarantine	7.24
3	tested positive, coronavirus outbreak, wuhan virus, positive coronavirus, confirmed cases, new york, shelter place, mental health, china virus, feel like, new cases, gt gt, coronavirus covid, virus, weeks, people virus, people don’t, bringing total, press conference, sars cov	8.87
4	dont think, virus spread, lockdown period, fake news, nursing homes, wuhan lab, best thing, months, lockdown amp, 21 3, id like, people know, real time, entire world, know im, know it, wake up, feel free, dont wanna, anthony fauci	6.56
5	u s, coronavirus cases, public health, save lives, novel coronavirus, long term, south korea, dont forget, bbc news, care homes, news coronavirus, million people, doesnt mean, family members, want know, coronavirus vaccine, going on, rest world, coronavirus, new jersey	7.36
6	at home, stay at, home order, thank you, look like, good news, test positive, people stay, fight virus, people protesting, face mask, good thing, young people, lock down, wearing masks, cases deaths, trump said, deaths reported, shut down, active cases	7.36
7	social distancing, day quarantine, healthcare workers, prime minister, world health, dont care, global pandemic, dont understand, health organization, dr fauci, let know, time lockdown, virus isn’t, in place, anti lockdown, shelter in, people think, live updates, 2 months	7.81
8	coronavirus lockdown, coronavirus crisis, amid coronavirus, looks like, new coronavirus, task force, im sure, coronavirus patients, prevent spread, virus doesn’t, dont let, long time, new York, high risk, coronavirus task, thank god, number deaths, dont like, virus outbreak, coronavirus cases	7.47
9	stay safe, chinese virus, self quarantine, need know, people going, new virus, common sense, safe stay, virus amp, b c, 2 2, family friends, we’ve got, got virus, stay away, testing kits, health amp, virus gone, april 20, knew virus	7.07
10	corona virus, new cases, death toll, im going, quarantine day, people died, spread coronavirus, cases coronavirus, people dying, quarantine im, total number, number cases, cases reported, april 2020, confirmed cases, coronavirus death, 24 hours, people need, stop spread	8.84
11	stay home, home orders, president trump, social media, home stay, loved ones, stay safe, death rate, working home, 31 000, social distance, 3100 000, protesting stay, breaking news, deaths, im sorry, 10 000, mortality rate	8.67
12	coronavirus pandemic, year old, united states, wash hands, people like, work home, god bless, lot people, wear mask, years ago, virus hoax, like virus, 23 days, grocery store, said virus, 21 million, watch video, 10 days, like amp, uk lockdown	7.06
13	right now, dont want, 3 weeks, tests positive, donald trump, weeks ago, weeks lockdown, virus spreading, coronavirus update, new zealand, 22 million, sounds like, total cases, lockdown 2, communist party, day day, chinese communist, cases 1, whats happening, 2 weeks	7.18

### COVID-19–Related Themes

The thematic analysis enabled us to categorize these topics into different distinct themes. The team considered the identified topics, bigrams, and representative tweet samples in each topic and categorized them into different themes. To protect the privacy and anonymity of the Twitter users, we did not present any user-related information, such as users’ Twitter handles or other identifying information. Therefore, sample tweets were excerpts drawn from original tweets in Table 3.

We organized 13 topics into 5 themes: “Public health measures to slow the spread of COVID-19” (eg, face masks, test kits, vaccine), “Social stigma associated with COVID-19” (eg, Chinese virus, Wuhan virus), “Coronavirus news cases and deaths” (eg, new cases, deaths), “COVID-19 in the United States” (eg, New York, protests, task force), and “Coronavirus cases in the rest of the world” (eg, UK, global issue). For example, the theme “public health measures to slow the spread of COVID-19” included the relevant topics of “facemasks,” “quarantine,” “test kits,” “lockdown,” “safety,” “vaccine,” and “shelter-in-place.” In addition, “home quarantine” and “self-quarantine” were two of the most commonly co-occurred words under the topic quarantine.

**Table 3:** Themes based on topic classification, bigrams, and sample tweets.

Theme and topic		Bigrams	Sample tweets
**Public health measures to slow the spread of COVID-19**			
	Face masks	face masks, wear masks	We protect us and our family by wearing masks every day.
	Quarantine	home quarantine, self quarantine, quarantine period	@realDonaldTrump @JustineTrudeau They’re all under mandatory 2 week quarantine, and they are essential workers…
	Test kits	test kits, testing kits	Hydroxychloroquine, Testing Kits and USA: We urge the Modi govt to draw proper lessons from this latest instance of US
	Lockdown	covid19 lockdown, lockdown period, weeks lockdown,	People are actually shocked the lockdown has been extended for 3 weeks when there are still people going out meeting
	Safety	stay safe, safe stay, stay away	Be strong, stay safe #lockdown but not locked out http://t.co/FvifiEbbs7
	Vaccine	coronavirus vaccine	Lead scientist for NIH working on #coronavirus vaccine research
	US shelter-in-place	Shelter place, shelter in	Did California’s shelter-in-place order work? If you sue crap data without any reference to epidemiology, then yes
**Social stigma associated with COVID-19**			
	Chinese Communist Party	communist party, Chinese communist, cases 1	The #Chinese Communist Party (#CCP) is spreading disinformation to cover up the origin of the #coronavirus
	Discriminatory names	Wuhan virus, Chinese virus	That China is responsible for putting entire world @great risk. Heavily criticized their eating habits.
	President Trump tweeting “Chinese virus”	president trump, social media, china virus	President Trump: They know where it came from. We all know where it came from, #chinesevirus
**COVID-19 new cases and deaths**			
	New cases	new cases, total number, confirmed cases	RT @neeratanden: 4,591 people died in a day from the virus, the highest number anywhere ever that we know of.
	Deaths	coronavirus death, death toll, people died	#Britain's death toll could be DOUBLE official tally as care homes
**COVID-19 in the United States**			
	Mental health and COVID-19 in New York	new york, shelter place, mental health	New Yorkers on their apartment roofs during quarantine is a whole different vibe. This is gonna be in history books
	Protests against the lockdown	anti lockdown, people protesting, protesting stay	I stand with the Healthcare workers!!! Bravo! Healthcare workers face off against anti-lockdown protesters in Colorado
	Task force in the United States	task force	RT @Jim_Jordan: There are #coronavirus task forces doing great work. But there is one task force that’s missing in action: the U.S. congress
	COVID-19 pandemic in the United States	united states, white house, new jersey, 21 million, million people, dr fauci,	Stay-at-home orders continue in much of the United States
**COVID-19 cases in the rest of the world**			
	United Kingdom	Herd immunity, UK lockdown, Prime Minister	The Prime Minister gave the game away early on when he openly said to Scrofulous and Willibooby that the government’s plan was Herd Immunity the REAL people in charge must have been so furious with him he had to be sent to an isolation ward with the virus to shut him up!
	Global issue	Entire world, south Korea, world health, global pandemic, new Zealand	Worldwide it is now 182,726.” And “New Zealand Prime Minster Jacinda Ardern says the government will partially relax its lockdown in a week, as a decline in …

### Sentiment Analysis

**Figure 6:**
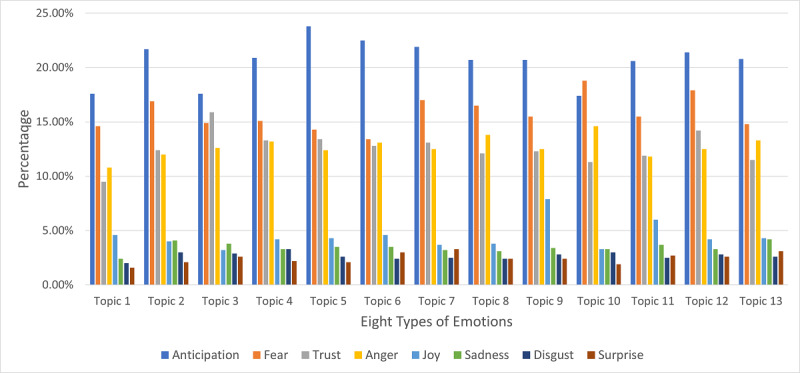
Sentiment analysis for each of the 13 latent topics.

We presented the results of the sentiment analysis for each of the 13 latent topics in Figure 6 and Table 4. Figure 6 presented 8 emotions of trust, anticipation, joy, surprise, anger, fear, disgust, and sadness. Results showed that across all 13 topics, anticipation (dark blue line) dominated 12 topics, followed by fear (orange line), trust (grey line), and anger (yellow line).

We also ran a one-tailed *z* test to examine if each of the 8 emotions is statistically significantly different across topics. A *P* value <.01 was set as the threshold for significance. For example, about 23.8% of tweets in Topic 5 revealed feelings of anticipation that “necessary steps and precautions will be taken” [18,29]. Statistical significance indicated that it was very likely (*P*<.001) that the anticipation emotion is more prevalently expressed in Topic 5 (23.8%) than all other topics. The emotion fear (of the impacts of the virus) was found in 18.8% of the tweets in Topic 10, which was statistically different from the fear expressed in other topics.

**Table 4:** The percentage of 8 emotions across 13 topics^a^.

Topic	Anger, %	Anticipation, %	Disgust, %	Fear , %	Joy, %	Sadness, %	Surprise, %	Trust, %
1	10.80	17.60	2.00	14.60	4.60^b^	2.40	1.60	9.50
2	12.00	21.70^b^	3.00^b^	16.90^b^	4.00	4.10^b^	2.10	12.40^b^
3	12.60^b^	17.60	2.90^b^	14.90	3.20	3.80^b^	2.60^b^	15.90^b^
4	13.20^b^	20.90^b^	3.30^b^	15.10	4.20	3.30^b^	2.20^b^	13.30^b^
5	12.40^b^	23.80^b^	2.60^b^	14.30	4.30	3.50^b^	2.10	13.40^b^
6	13.10^b^	22.50^b^	2.40	13.40	4.60^b^	3.50^b^	3.00^b^	12.80^b^
7	12.50^b^	21.90^b^	2.50^b^	17.00^b^	3.70	3.20^b^	3.30^b^	13.10^b^
8	13.80^b^	20.70^b^	2.40	16.50^b^	3.80	3.10^b^	2.40^b^	12.10^b^
9	12.50^b^	20.70^b^	2.80^b^	15.50	7.90^b^	3.40^b^	2.40^b^	12.30^b^
10	14.60^b^	17.40	3.00^b^	18.80^b^	3.30	3.30^b^	1.90	11.30
11	11.80	20.60^b^	2.50^b^	15.50^b^	6.00^b^	3.70^b^	2.70^b^	11.90^b^
12	12.50^b^	21.40^b^	2.80^b^	17.90^b^	4.20	3.30^b^	2.60^b^	14.20^b^
13	13.30^b^	20.80^b^	2.60^b^	14.80	4.30	4.20^b^	3.10^b^	11.50^b^

^a^The sum of the percentages for each topic is not equal to 100%. The remainder is made up of neutral or other emotions.

^b^*P*<.001 from *z* test.

## Discussion

### Principal Results

In this study, we addressed public discussions and emotions using COVID-19–related messages on Twitter. Twitter users discussed 5 main themes related to COVID-19 between March 7 and April 21, 2020. Topic modeling of the tweets was useful for providing insights about COVID-19 topics and concerns. Results showed several essential points. First, the public uses a variety of terms when referring to COVID-19, including virus, COVID-19, coronavirus, and corona virus. Second, COVID-19 has been referred to as the “China virus,” which can create stigma and harm efforts to address the COVID-19 outbreak [14]. Third, discussions about the pandemic in New York were salient, and its associated public sentiment was anger. Fourth, public discussions about the Chinese Communist Party (CCP) and the spread of the virus emerged as a new topic that was not identified in previous studies [18], suggesting the connection between COVID-19 and politics is increasingly circulating on Twitter as the situation evolves. Fifth, public sentiments on the spread of COVID-19 reveal anticipation for the potential measures that can be taken, followed by mixed feelings of trust, anger, and fear. Results suggest that the public is not surprised by the rapid spread of COVID-19. Sixth, people have a significant feeling of fear when they discuss the COVID-19 crisis and deaths. Lastly, trust is no longer a prominent emotion when Twitter users discuss COVID-19, which is different from the findings of an earlier study [18].

### Comparison With Prior Work

Our findings are consistent with previous studies using social media data to assess the public health responses and sentiments related to COVID-19, and suggest that public attention has been focusing on the following topics since January 2020: (1) the confirmed cases and death rates [11,18,30], (2) preventive measures [11,18,31], (3) health authorities and government policies [10,18], (4) an outbreak in New York [18], (5) COVID-19 stigma (eg, referring to COVID-19 as the “Chinese virus”) [11,14], and (6) negative psychological reactions (eg, fear) or mental health consequences [11,31-33].

Compared with a study examining public discussions and concerns related to COVID-19 using Twitter data from January 20 to March 7, 2020, we found that several salient topics are no longer popular: (1) an outbreak in South Korea, (2) the *Diamond Princess* cruise ship, (3) the economic impact [11,32], and (4) supply chains [18]. Given current preventive measures, washing hands is no longer a prevalent topic; instead, quarantine has become dominant.

In addition, our study identified new discussion topics about COVID-19 occurring between March 7 to April 21: (1) the need for a vaccine to stop the spread, (2) quarantine and shelter-in-place orders, (3) protests against the lockdown, and (4) the COVID-19 pandemic in the United States. The new salient topics suggest that Twitter users (tweeting in English) are focusing their attention on COVID-19 in the United States (eg, New York, protests, task force, millions of confirmed cases) rather than global news (eg, South Korea, *Diamond Princess* cruise ship, Dr Li Wenliang in China).

### Limitations

First, we only sampled 20 hashtags as the key search terms to collect Twitter data (Multimedia Appendix 1). New hashtags keep coming up as the situation evolves. For example, a hashtag may become widely used after a related topic becomes more popular, such as the official name for the virus (COVID-19). Second, Twitter users are not representative of the whole global population, and topics of tweets only indicate online users' opinions about and reactions to COVID-19. However, the Twitter data set is still a valuable resource, allowing us to examine real-time Twitter users’ responses and online activities related to COVID-19. Third, non-English tweets were removed from our analyses, and hence the results are limited to users who posted in English only. Future COVID-19 studies should include other languages, such as Italian, French, German, and Spanish.

### Future Research

Future research could further explore public trust and confidence in existing measures and policies, which are essential. Compared to prior work, our study showed that Twitter users had a feeling of joy when talking about herd immunity. Sentiments of fear and anticipation related to the topics of quarantine and shelter-in-place. Future studies could evaluate how government officials (eg, President Trump) and international organizations (eg, World Health Organization) deliver and convey messages to the public, and the subsequent impact on public opinions and sentiments. Anti-Chinese/Asian sentiments spread on social media, and it would be worth assessing how people use these platforms to resist and challenge COVID-19 stigma. Misinformation during the COVID-19 pandemic was not a prominent theme in this study. An existing study showed that 25% (n=153) of sampled tweets contained misinformation [34]. The term COVID-19 has lower rates of misinformation associated with it than that associated with #2019_ncov and Corona. Future research should investigate misinformation and how it expands on social media. Finally, trust is no longer prominent when people tweet about confirmed cases and deaths. Instead, fear has replaced trust to be the dominant emotion. Future research should examine the changes in trust over time.

### Conclusions

Twitter data and machine learning approaches can be leveraged for infodemiology studies by studying evolving public discussions and sentiments during the COVID-19 pandemic. Our findings facilitate an understanding of public discussions and concerns about the COVID-19 pandemic among Twitter users between March 7 and April 21, 2020. Several topics were consistently dominant on Twitter, such as “the confirmed cases and death rates,” “preventive measures,” “health authorities and government policies,” “stigma,” and “negative psychological reactions” (eg, fear). As the situation rapidly evolves, new salient topics emerge accordingly. Fear arises in messages of new cases or death reports [18]. Real-time monitoring and assessment of Twitter users’ concerns can be promising for informing public health emergency responses and planning. Hearing and reacting to real concerns from the public can enhance trust between the health care system and the public and enable better preparation for a future public health emergency.

## Appendix

Multimedia Appendix 1 Supplementary data.

## Abbreviations

API: Application Programming Interface
CCP: Chinese Communist Party
LDA: Latent Dirichlet Allocation
NLP: Natural Language Processing
