# Supplementary material for: Twitter Discussions and Emotions About the COVID-19 Pandemic: Machine Learning Approach
Source: J Med Internet Res. 2020 Nov 25;22(11):e20550. doi: 10.2196/20550 (PMC7690968; doi:10.2196/20550)
Supplement: Multimedia Appendix 1 [file jmir_v22i11e20550_app1.docx]

Appendix

Appendix 1. The list of hashtags used as search terms for data collection

| #COVID19 | #Covid19 | #covid19 | #Covid_19 | #COVID |
| --- | --- | --- | --- | --- |
| #coronavirus | #Coronavirus | #CoronaVirus | #2019nCoV | #CoronavirusOutbreak |
| #StayHome | #stayhome | #Lockdown | #lockdown | #CoronavirusPandemic |
| #Qurantine | #qurantine | #2019nCoV | #FireTrump | #SARsCov2 |
